# Metachronous triple cancers of the stomach, duodenum and rectum in a patient with familial adenomatous polyposis

**DOI:** 10.1093/jscr/rjac508

**Published:** 2022-11-15

**Authors:** Shuichiro Uchiyama, Naotaka Ikeda, Tomohiro Oyama, Mayumi Eguchi, Rikiya Sato, Ayaka Ito, Ryouichi Toyosaki, Masaki Kitazono, Toyokuni Suenaga

**Affiliations:** Department of Surgery, Nanpuh Hospital, Kagoshima, Japan; Department of Surgery, Nanpuh Hospital, Kagoshima, Japan; Department of Surgery, Nanpuh Hospital, Kagoshima, Japan; Department of Surgery, Nanpuh Hospital, Kagoshima, Japan; Department of Surgery, Nanpuh Hospital, Kagoshima, Japan; Department of Surgery, Nanpuh Hospital, Kagoshima, Japan; Department of Surgery, Nanpuh Hospital, Kagoshima, Japan; Department of Surgery, Nanpuh Hospital, Kagoshima, Japan; Department of Surgery, Nanpuh Hospital, Kagoshima, Japan

## Abstract

Familial adenomatous polyposis (FAP) is an autosomal dominant disorder characterized by the presence of at least 100 adenomatous polyps in the colon and rectum. The risk of upper gastrointestinal tumors is relatively high in patients with FAP, but a case of triple cancers has not been reported in the literature. We herein report a case of metachronous triple cancers of the stomach, duodenum and rectum in a patient with FAP.

## INTRODUCTION

Familial adenomatous polyposis (FAP) is characterized by the presence of at least 100 adenomatous polyps in the colon and rectum [1]. Upper gastrointestinal carcinoma is reported in ~5% of patients with FAP, but triple cancers in patients with FAP have not been reported so far. We describe the occurrence and treatment of metachronous triple cancers of the stomach, duodenum and rectum in a patient with FAP.

## CASE REPORT

**Figure 1:**
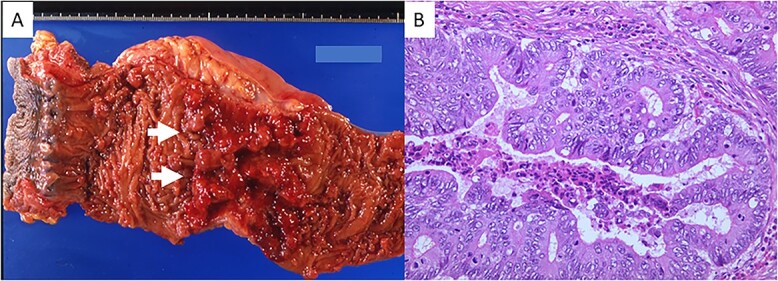
(**A**) Gross appearance of the resected portion of the rectum. A Type 2 tumor (white arrows) is seen. (**B**) Microscopic examination revealed well-differentiated adenocarcinoma (hematoxylin and eosin stain; original magnification, ×200).

**Figure 2:**
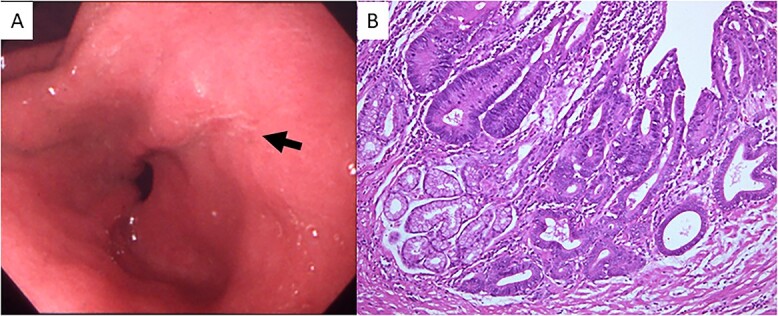
(**A**) Upper gastrointestinal endoscopy showed a depressed lesion (black arrow) in the posterior wall of the antrum. (**B**) Microscopic examination revealed proliferation of atypical cells forming irregular tubular structures (hematoxylin and eosin stain; original magnification, ×100).

**Figure 3:**
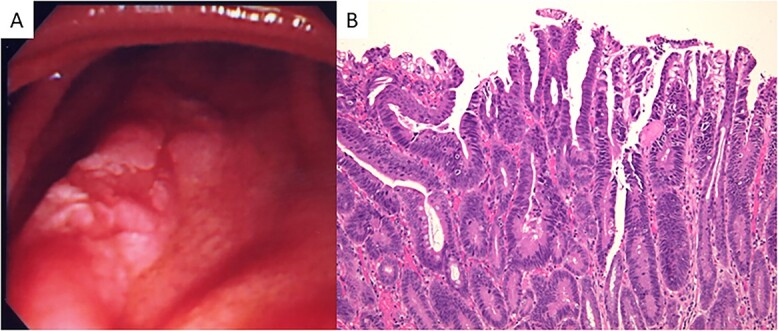
(**A**) Endoscopic appearance of the duodenal lesion. A IIa + IIc lesion is seen. (**B**) Microscopically, a well-differentiated adenocarcinoma is seen (hematoxylin and eosin stain; original magnification, ×100).

A 23-year-old man with a complaint of constipation and narrow stool was diagnosed as FAP by a barium enema study in 1984, and an ulcerated tumorous lesion was detected at the rectum. There was no known family history of FAP. The patient was admitted to our hospital, and a colonoscopy revealed a Type 2 tumor in the lower rectum. Numerous polypoid lesions were observed from the transverse colon to the rectum, but only a few polypoid lesions were seen at the cecum and ascending colon. Subtotal colectomy, abdominoperineal dissection and colostomy were performed, and the ascending colon and cecum were preserved. Numerous polypoid lesions, up to ~1 cm in diameter, were seen in the resected specimen. A circular, well-demarcated, ulcerated lesion, 6.0 × 8.5 cm in size, was seen at the rectum 6 cm from the anal verge (Fig. 1A). Histologic examination of the tumor revealed a well-differentiated tubular adenocarcinoma (Fig. 1B) invading the subserosal layer accompanying both lymphatic and vascular invasion. There was no lymph node metastasis. The number of polypoid lesions gradually increased in the residual colonic mucosa, and, resection of the residual colon and ileostomy was performed in 1998, 14 years after the first surgery. No cancerous lesion was seen in the resected specimen upon histologic examination. Fourteen months after total colectomy, a depressed lesion (Fig. 2A) was detected in the posterior wall of the antrum in addition to a benign duodenal ulcer with the upper gastrointestinal endoscopy. Histologic examination of the depressed lesion revealed an adenocarcinoma component. There was another depressing lesion in the gastric angle showing histologic mild structural atypia, so distal partial gastrectomy with regional lymph node dissection was performed. An IIa + IIc type depressed lesion, 5 mm in diameter, was seen at the antrum of the resected specimen. Histologic examination showed well-differentiated tubular adenocarcinoma, limited to the mucosal layer (Fig. 2B). Neither lymphatic nor vascular invasion was seen, and there was no lymph node metastasis. Another lesion at the angle was slightly depressed, 5 mm in diameter, and histologic examination revealed the atypical glands with moderate dysplasia but no convincing evidence of malignancy. In 2001, 16 months after distal partial gastrectomy, a follow-up upper gastrointestinal endoscopy revealed a superficial elevated lesion with central depression (IIa + IIc) in the proximal third portion of the duodenum (Fig. 3A). Histologic examination of the endoscopic biopsy specimen of the lesion revealed a well-differentiated adenocarcinoma component. An adenomatous focus was seen in the mucosa adjacent to the cancerous lesion. Endoscopic mucosal resection was performed for this lesion. The tumor cells proliferated in a tubular fashion, suggesting well-differentiated adenocarcinoma (Fig. 3B), and were confined to the mucosal layer. There was neither apparent lymphatic nor vessel invasion, and the resected margin was free of cancer cells. Because the patient rejected genetic testing, the adenomatous polyposis coli (APC) gene mutation is unclear.

## DISCUSSION

Approximately 75% of FAP patients have an inherited mutation in the APC gene on the long arm of chromosome 5 (5q21-22) and have a parent and possibly other relatives with the mutation. The APC gene itself is very large, and the reported mutations now number >300. Screening for mutations is difficult because of their distribution throughout the gene and the size of the gene [2].

It is widely known that upper gastrointestinal tumors including benign and malignant lesions occur at an increased rate in patients with FAP. Church *et al*. investigated the prevalence of gastroduodenal polyps in 147 patients with FAP and found duodenal adenomas in 88% of patients and fundic gland polyps in 84% [3]. Jagelman *et al*. reported that invasive upper gastrointestinal adenocarcinoma was found in 4.5% of 1255 patients with FAP. Specific incidences were as follows: gastric cancer 0.56%, duodenal cancer 2.3% and ampullary cancer 0.8% [4]. Histopathologic analysis of duodenal cancer in patients with FAP was performed by Spigelman *et al.*, and adenomatous tissue was found within duodenal cancer in 66% and mucosa adjacent to duodenal cancer in 73% of patients. Adenomas were found as a component of, or adjacent to, duodenal cancer in 84% of patients, suggesting the adenoma-carcinoma sequence in these patients [5]. The adenomatous residue was found in the mucosa adjacent to the duodenal lesion in our case; thus, there is some possibility that the adenoma-carcinoma sequence was involved in our case. Offerhaus *et al*. found no significant increase in the risk of gastric or nonduodenal small intestinal cancer in patients with FAP, but they did find an increased risk of duodenal adenocarcinoma (relative risk, 330.82) and ampullary adenocarcinoma (relative risk, 123.72; [6]).

Whether gastric adenocarcinoma in our case is related to FAP is unclear. There is an ~20-fold difference in the incidence of gastric carcinoma between Japanese and some Caucasian populations in the USA. Gastric carcinoma also occurs in patients with gastrointestinal polyposis syndromes including FAP; however, gastric carcinoma is rare in these settings, and the exact contribution of the polyposis and underlying germline alterations of the APC gene to cancer development is unclear [7].

The necessity of screening FAP patients for upper gastrointestinal adenoma has been advocated. This is clinically difficult, however, because the size is no guide to the malignant potential, adenomatous changes can occur in macroscopically flat mucosa, and biopsy of a single lesion is likely to miss a malignant change in ~50% of cases [8].

As far as we know, there is no other report of metachronous triple cancers in different organs in a patient with FAP. Careful long-term follow-up for detection of newly developed malignant gastroduodenal neoplasms is required in patients with FAP, even after total colectomy.

## References

[1] Tomita N, Ishida H, Tanakaya K, Yamaguchi T, Kumamoto K, Tanaka T, et al. Japanese Society for Cancer of the Colon and Rectum (JSCCR) guidelines 2020 for the Clinical Practice of Hereditary Colorectal Cancer. Int J Clin Oncol 2021;26:1353–419. 34185173 10.1007/s10147-021-01881-4 PMC8286959

[2] Bronner MP . Gastrointestinal inherited polyposis syndromes. Mod Pathol 2003;16:359–65. 12692201 10.1097/01.MP.0000062992.54036.E4

[3] Church J, Burke C, McGannon E, Pastean O, Clark B. Risk of rectal cancer in patients after colectomy and ileorectal anastomosis for familial adenomatous polyposis: a function of available surgical options. Dis Colon Rectum 2003;46:1175–81. 12972960 10.1007/s10350-004-6710-2

[4] Jagelman DG, DeCosse JJBH. Upper gastrointestinal cancer in familial adenomatous polyposis. Lancet 1988;1:1149–51. 2896968 10.1016/s0140-6736(88)91962-9

[5] Spigelman AD, Talbot IC, Penna C, Nugent KP, Phillips RK, Costello C, et al. Evidence for adenoma-carcinoma sequence in the duodenum of patients with familial adenomatous polyposis. The Leeds Castle Polyposis Group (Upper Gastrointestinal Committee). J Clin Pathol 1994;47:709–10. 7962621 10.1136/jcp.47.8.709 PMC502141

[6] Offerhaus GJA, Giardiello FM, Krush AJ, Booker SV, Tersmette AC, Kelley NC, et al. The risk of upper gastrointestinal cancer in familial adenomatous polyposis. Gastroenterology 1992;102:1980–2. 1316858 10.1016/0016-5085(92)90322-p

[7] LA HSR-A . World Health Organization Classification of Tumours. Pathology and Genetics of Tumours of the Digestive System. Lyon, France: IARCPress, 2000.

[8] Rhodes M, Bradburn DM. Overview of screening and management of familial adenomatous polyposis. Gut 1992;33:125–31. 1310949 10.1136/gut.33.1.125 PMC1373878

